# Perioperative Aspirin Continuation in Patients Requiring Secondary Prevention Undergoing Anatomical Lung Resection: A Propensity Score‐Weighted Analysis

**DOI:** 10.1111/1759-7714.70364

**Published:** 2026-07-21

**Authors:** Junichi Murakami, Sota Yoshimine, Mototsugu Shimokawa, Toshiki Tanaka

**Affiliations:** ^1^ Department of Surgery and Clinical Science, Division of Chest Surgery Yamaguchi University Graduate School of Medicine Yamaguchi Japan; ^2^ Department of Biostatistics Yamaguchi University Graduate School of Medicine Yamaguchi Japan; ^3^ Department of Data Science, Center for Clinical Research Yamaguchi University Hospital Ube Japan

## Abstract

**Background:**

Managing perioperative aspirin for secondary prevention during lung resection balances major bleeding against catastrophic thromboembolism. Evidence guiding this scenario, especially for real‐world strategies involving switches from other antiplatelets, remains limited for anatomical resections.

**Methods:**

This retrospective study (2018–2025) included 625 patients undergoing anatomical lung resection. We compared aspirin continuation (Group 1, *n* = 94; chronic users or clopidogrel switchers) with no‐therapy controls (Group 3, *n* = 468). Inverse probability of treatment weighting (IPW) was used to balance 16 predefined covariates. The discontinuation group (Group 2, *n* = 63) served as a descriptive clinical reference. Primary endpoints were major bleeding (reoperation, ≥ 4 units RBC transfusion, or death) and major adverse cardiac and cerebrovascular events (MACCEs) within 30 days.

**Results:**

After IPW adjustment, all covariates achieved satisfactory balance (SMD < 0.1). Group 1 showed a potential trend toward increased major bleeding (odds ratio [OR], 5.87; 95% confidence interval [CI], 0.80–42.91; *p* = 0.08), but no significant difference in MACCE (OR, 1.51; 95% CI, 0.35–6.45; *p* = 0.58) compared to Group 3. In adjusted linear regression, aspirin was not an independent predictor of intraoperative blood loss (*p* = 0.25). Notably, Group 2 (discontinuation) exhibited alarmingly high rates of MACCE (7.9%) and 90‐day mortality (4.8%). Analyses for rare events were statistically underpowered.

**Conclusion:**

Continuing aspirin showed a nonsignificant trend toward increased major bleeding, so its overall safety remains uncertain. However, the high event rates after stopping therapy highlight the significant risk of thromboembolism. Clinical judgment must carefully balance individual thrombotic benefits with potential bleeding risks.

## Graphical abstract

In 625 patients undergoing anatomical lung resection, we compared perioperative aspirin continuation with no antithrombotic therapy using propensity‐score (IPW) weighting. Continuing aspirin showed only a nonsignificant trend toward major bleeding (OR 5.87, *p* = 0.08) and no increase in cardiac or cerebrovascular events. Conversely, patients who stopped therapy had notably high MACCE (7.9%) and 90‐day mortality (4.8%), highlighting the thrombotic risk of interruption.

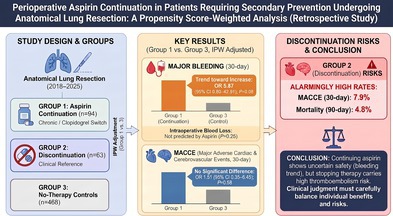

## 1 Introduction

The perioperative management of low‐dose aspirin in patients undergoing major surgery presents a critical clinical dilemma, balancing the risk of surgical bleeding against the risk of catastrophic thromboembolic events. Discontinuing aspirin in high‐risk, secondary‐prevention patients may increase the risk of myocardial infarction or stroke, yet continuing it may worsen intraoperative bleeding [1, 2].

Large randomized controlled trials, such as the POISE‐2 trial [3], found that aspirin significantly increased major bleeding without reducing cardiovascular events in a broad non‐cardiac surgery population. Based on these findings, current guidelines recommend stopping aspirin for primary prevention [4, 5]. Still, the optimal strategy for high‐risk secondary prevention patients, who were often excluded from these trials, remains highly contentious. Furthermore, high‐quality evidence specifically evaluating aspirin continuation in the context of anatomical lung resection remains limited. This lack of specific data for thoracic surgery, a high‐stakes field with unique comorbidities, has led to considerable practice variation and highlights a clear need for dedicated research.

Therefore, the primary goal of this retrospective study was to assess the real‐world, short‐term outcomes of a perioperative “aspirin‐continuation strategy,” which included high‐risk patients switched from clopidogrel. To quantify the additional risk, we used inverse probability weighting (IPW) based on propensity scores to compare these high‐risk patients with a control group without a history of antithrombotic therapy.

## 2 Methods

### 2.1 Study Design and Patient Population

This retrospective, single‐center cohort study was conducted at Yamaguchi University Hospital and approved by the Yamaguchi University Research Ethics Committee (IRB number: H2025‐090), which waived patient consent due to the study's retrospective, anonymized nature. We identified all consecutive adult patients (age ≥ 20, Eastern Cooperative Oncology Group Performance Status [ECOG‐PS] ≤ 3) who underwent elective lung resection for malignant conditions between January 2018 and June 2025. Exclusion criteria were planned open thoracotomy, non‐anatomical resections, resections more than bilobectomy, sleeve resections, and simultaneous bilateral surgery. Patients undergoing anatomical resection with a concomitant ipsilateral wedge resection were included. Cases involving ≥ 2 different procedures were defined as ‘concomitant lung resection’.

### 2.2 Group Definition

Patients were divided into three groups. The Aspirin‐continuation group (Group 1) included patients who maintained perioperative aspirin; this group comprised those continuing chronic low‐dose aspirin (100 mg) for secondary prevention and those switched from chronic clopidogrel to aspirin perioperatively. The control group (Group 3) consisted of patients with no history of antithrombotic therapy. A third group (Group 2), consisting of patients on any antiplatelet or anticoagulant treatment that was discontinued perioperatively, was also identified. Data from Group 2 are presented descriptively but were excluded from formal comparative analysis due to the limited sample size and significant heterogeneity among discontinued agents. This group serves as a clinical reference to illustrate the potential risks of interrupting antithrombotic therapy in a real‐world setting.

### 2.3 Data Collection and Definition

Clinical data, including demographics, surgical outcomes, and postoperative information, were retrospectively gathered from institutional electronic medical records. The primary endpoints were the occurrence of major bleeding and major adverse cardiac and cerebrovascular events (MACCEs) within 30 days. Major bleeding was defined as a combination of reoperation for bleeding, red blood cell transfusion of ≥ 2 units within 48 h, or bleeding that resulted in death. MACCE included all‐cause mortality, nonfatal myocardial infarction, and nonfatal ischemic stroke. Secondary endpoints encompassed intraoperative blood loss, surgical duration, chest tube drainage time, hospital stay length, and complications graded ≥ II by the Clavien–Dindo classification.

### 2.4 Perioperative Antiplatelet and Anticoagulant Management

Preoperative antithrombotic therapy was managed via institutional protocols, individualized to each patient's thrombotic and hemorrhagic risk. In the discontinuation group (Group 2), antiplatelet agents (e.g., clopidogrel, cilostazol) were typically stopped 7–14 days pre‐surgery. Warfarin was stopped 7 days before surgery, and direct oral anticoagulants (DOACs) were stopped 2 days before surgery, often replaced by heparin bridging. Intravenous heparin was discontinued 4 h preoperatively. Anticoagulant therapy resumed post‐chest tube removal once hemostasis was confirmed.

### 2.5 Statistical Analysis

All analyses used STATA version 19 (StataCorp LLC, College Station, TX, USA) with *p* < 0.05 indicating significance. As continuous variables lacked normality, they were reported as medians (IQRs); categorical variables were reported as *n* (%). For descriptive (three‐group) comparisons, the Kruskal–Wallis test (for continuous) and chi‐squared or Fisher's exact test (for categorical) were used. Significant overall results prompted post hoc Dunn tests (Bonferroni correction) or pairwise comparisons with Bonferroni‐adjusted *p* values, respectively.

To address selection bias between the aspirin‐continuation and Control groups, we performed a propensity score analysis. A multivariable logistic regression model, including 16 predefined covariates (age, gender, bmi, smoking history, % predicted VC, FEV_1.0_/FVC, interstitial pneumonia, surgical approach, operative procedure, complex segmentectomy, concomitant lung resection, resected side, resected lobe, nodal dissection, whole pleural adhesion, and incomplete pulmonary fissure), estimated the propensity score. We then calculated IPW. Covariate balance was assessed using the standardized mean difference (SMD), with < 0.1 deemed negligible. In the IPW‐adjusted cohort, we compared outcomes using weighted logistic (binary) and weighted linear (continuous) regression.

As a secondary analysis, a multivariable linear regression model identified independent predictors of intraoperative blood loss (Groups 1 and 3 only). Clinically relevant variables were screened using univariate regression; those with *p* < 0.2, along with group allocation, entered the final multivariable model.

## 3 Results

### 3.1 Patient Selection and Baseline Characteristics

**FIGURE 1:**
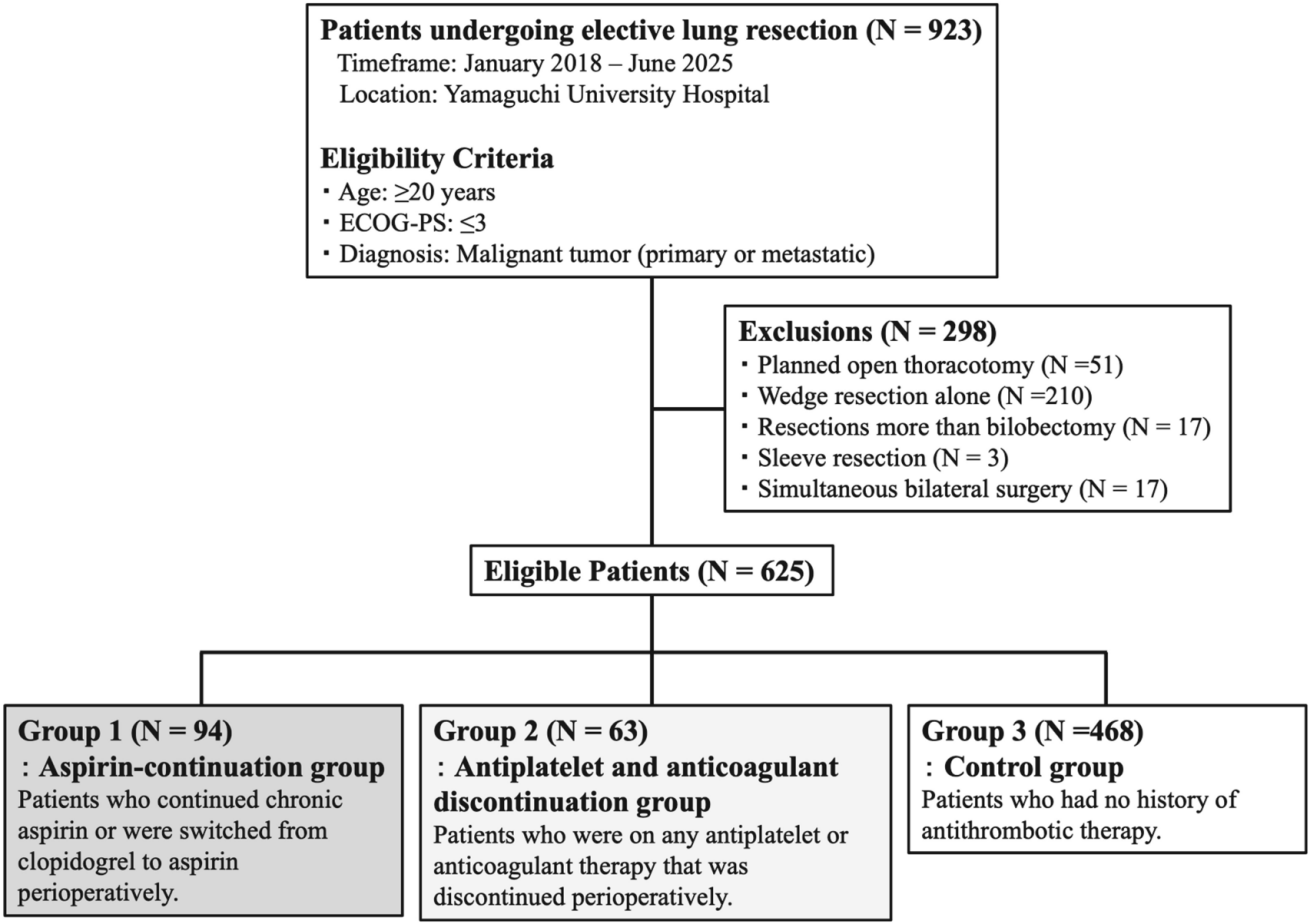
Patient selection flowchart. A total of 923 patients undergoing elective lung resection were screened. After excluding 298 patients based on the specified criteria, 625 patients were included in the final analysis and stratified into three groups.

As shown in Figure 1, 923 patients underwent elective lung resection during the study period. After excluding 298 patients who met the exclusion criteria, 625 patients were eligible for the final analysis. These patients were stratified into the aspirin‐continuation group (Group 1, *n* = 94), the antiplatelet‐ and anticoagulant‐discontinuation group (Group 2, *n* = 63), and the control group (Group 3, *n* = 468).

The baseline patient and surgical characteristics for all three groups are presented in Table 1. As specified in the statistical methods, differences among the three groups were assessed, and post hoc Bonferroni‐adjusted comparisons were performed between Group 1 and Group 3. Before adjustment, patients in the Aspirin‐continuation group were significantly older and more frequently male. They had a higher prevalence of comorbidities, including hypertension, diabetes mellitus, interstitial pneumonia, ischemic heart disease, and cerebrovascular disease, compared to the control group (all *p* < 0.05). Furthermore, patients in Group 1 had significantly lower baseline pulmonary function, including %FEV_1.0_ and FEV_1.0_/FVC (all *p* < 0.01). The specific details of preoperative antithrombotic agents for Group 1 and Group 2 are provided in Table S1.

**TABLE 1:** Baseline patient and surgical characteristics.

Variables	All patients	Group 1	Group 2	Group 3	*p* (among 3 groups)	Bonferroni‐adjusted *p* (Group 1 vs. 3)
		Aspirin‐continuation	Antiplatelet and anticoagulant discontinuation	Control		
	*n* = 625 [IQR] (%)	*n* = 94 [IQR] (%)	*n* = 63 [IQR] (%)	*n* = 468 [IQR] (%)		
Age (years)	73 [67–78]	76 [71–79]	76 [71–79]	72 [65–77]	< 0.001^a^	< 0.001^c^
Sex						
Male/female	367/258	73/21	43/20	251/217	< 0.001^b^	< 0.001^d^
Height (m)	1.61 [1.54–1.67]	1.62 [1.59–1.66]	1.62 [1.55–1.67]	1.60 [1.53–1.67]	0.27^a^	0.12^c^
BMI	22.7 [20.7–25.0]	23.2 [20.8–25.0]	23.3 [21.3–25.8]	22.5 [20.5–24.9]	0.25^a^	0.65^c^
ECOG‐PS						
0/1/2/3	572/44/7/2	82/11/0/1	57/6/0/0	433/27/7/1	0.17^b^	0.18^d^
Smoking history						
Yes/No	414/211	84/10	47/16	283/185	< 0.001^b^	< 0.001^d^
VC (% predicted)	104.1 [94.8–113.9]	101.3 [93.8–111.9]	105.2 [94.7–115.4]	104.3 [95.5–114.4]	0.18^a^	0.11
FEV_1.0_ (% predicted)	95.7 [83.9–107.7]	90.1 [81.0–100.8]	95.0 [83.7–107.7]	97.2 [84.6–108.9]	0.01^a^	0.004
FEV_1.0_/FVC (%)	71.7 [65.2–76.9]	69.9 [62.9–74.5]	70.3 [64.3–74.6]	72.3 [65.8–77.5]	0.005^a^	0.005
DLco (% predicted)	102.9 [81.4–126.8]	92.8 [73.3–111.2]	99.2 [81.4–125.6]	104.8 [83.3–128.2]	0.005^a^	0.002
Comorbidity (%)	568 (90.9)	94 (100.0)	62 (98.4)	412 (88.0)	< 0.001^b^	< 0.001^d^
Hypertension (%)	394 (63.0)	77 (81.9)	48 (76.2)	269 (57.5)	< 0.001^b^	< 0.001^d^
Dyslipidemia (%)	197 (31.5)	41 (43.6)	18 (28.6)	138 (29.5)	0.02^b^	0.02^d^
Diabetes mellitus (%)	141 (22.6)	35 (37.2)	18 (28.6)	88 (18.8)	< 0.001^b^	< 0.001^d^
Chronic obstructive pulmonary disease (%)	267 (42.7)	47 (50.0)	30 (47.6)	190 (40.6)	0.18^b^	0.28^d^
Interstitial pneumonia (%)	81 (13.0)	28 (29.8)	11 (17.5)	42 (9.0)	< 0.001^b^	< 0.001^d^
Treatment of malignancy (≤ 5 years ago) (%)	168 (26.9)	15 (16.0)	17 (27.0)	136 (29.1)	0.03^b^	0.03^d^
Aortic disease (%)	8 (1.3)	3 (3.2)	1 (1.6)	4 (0.9)	0.10^b^	0.19^d^
Arrhythmia (%)	47 (7.5)	5 (5.3)	31 (49.2)	11 (2.4)	< 0.001^b^	0.34^d^
Heart valve disease (≥ moderate) (%)	24 (3.8)	3 (3.2)	8 (12.7)	13 (2.8)	0.003^b^	0.83^d^
Ischemic heart disease (%)	56 (9.0)	46 (48.9)	2 (3.2)	8 (1.7)	< 0.001^b^	< 0.001^d^
Cerebrovascular disease (%)	73 (11.7)	32 (34.0)	25 (39.7)	16 (3.4)	< 0.001^b^	< 0.001^d^
Chronic kidney disease (Grade ≥ 3b)	82 (13.1)	29 (30.9)	11 (17.5)	42 (9.0)	< 0.001^b^	< 0.001^d^
Hemodialysis (%)	9 (1.4)	6 (6.4)	1 (1.6)	2 (0.4)	< 0.001^b^	< 0.001^d^
Liver cirrhosis (%)	3 (0.5)	0 (0.0)	0 (0.0)	3 (0.6)	0.60^b^	0.83^d^
Autoimmune disease	18 (2.9)	4 (4.3)	1 (1.6)	13 (2.8)	0.48^b^	0.79^d^
Hypothyroidism (%)	8 (1.3)	0 (0.0)	1 (1.6)	7 (1.5)	0.58^b^	0.70^d^
Oral steroids (%)	26 (4.2)	6 (6.4)	1 (1.6)	19 (4.1)	0.33^b^	0.86^d^
Tumor factors						
Primary disease						
Primary cancer/metastatic tumor	571/54	91/3	59/4	421/47	0.07^b^	0.10^d^
Tumor size (mm)	22 [16–30]	23 [17–30]	22 [17–31]	21 [16–31]	0.42^a^	0.39^c^
Consolidation size (mm)	16 [9–25]	21 [11–28]	17 [11–22]	16 [9–24]	0.21^a^	0.11^c^
SUV_max_ (FDG‐PET)	3.1 [1.4–7.4]	4.8 [1.5–10.4]	2.4 [1.3–5.6]	2.9 [1.4–7.2]	0.13^a^	0.10^c^
Pathological stage of primary cancer						
0/IA/IB/IIA/IIB/IIIA/IIIB/IV	63/318/111/25/16/32/2/5	6/50/23/5/0/5/0/2	6/33/12/3/3/2/0/0	51/235/76/17/13/25/2/3	0.59^b^	0.67^d^
Histologic subtype of primary cancer						
AD/SQ/others	440/85/46	53/25/13	49/7/3	338/53/30	< 0.001^b^	< 0.001^d^
Induction therapy	25 (4.0)	4 (4.3)	2 (3.2)	19 (4.1)	1.00^b^	0.93^d^
Surgical factors						
Surgical approach						
VATS/RATS	545/80	87/7	56/7	402/66	0.20^b^	0.24^d^
Operative procedure						
Lobectomy/segmentectomy	464/161	75/19	49/14	340/128	0.28^b^	0.45^d^
Complex segmentectomy (%)	73 (11.7)	11 (11.7)	5 (7.9)	57 (12.2)	0.62^b^	0.90^d^
Concomitant lung resection (%)	27 (4.3)	2 (2.1)	2 (3.2)	23 (4.9)	0.43^b^	0.70^d^
Resected side						
Right/left	380/245	56/38	38/25	286/182	0.96^b^	0.79^d^
Resected main lobe						
RUL/RML/RLL/LUL/LLL	217/40/126/148/94	33/7/18/22/14	19/3/16/11/14	165/30/92/115/66	0.72^b^	0.99^d^
Nodal dissection						
Hilar/hilar + mediastinal LNs	271/354	40/54	27/36	204/264	0.98^b^	0.85^d^
Pleural adhesion						
None/< 1/3/< 2/3/whole	368/211/29/17	52/33/4/5	44/16/3/0	272/162/22/12	0.35^b^	0.87^d^
Incomplete pulmonary fissure (%)	458 (73.3)	72 (76.6)	47 (74.6)	339 (72.4)	0.69^b^	0.86^d^

*Note:* Values are expressed as number (%) or median (interquartile range [IQR]).

Abbreviations: AD, adenocarcinoma; BMI, body mass index; CI, confidence interval; DLco, diffusing capacity of carbon monoxide; ECOG‐PS, Eastern Cooperative Oncology Group Performance Status; FDG‐PET, fluorodeoxyglucose‐positron emission tomography; FEV_1_, forced expiratory volume in 1 s; FVC, forced vital capacity; LLL, left lower lobe; LNs, lymph nodes; LUL, left upper lobe; RATS, robot‐assisted thoracoscopic surgery; RLL, right lower lobe; RML, right middle lobe; RUL, right upper lobe; SQ, squamous cell carcinoma; SUV_max_, standardized uptake value maximum; VATS, video‐assisted thoracoscopic surgery; VC, vital capacity.

^a^ Compared by the Kruskal–Wallis test.

^b^ Compared using the chi‐square test or Fisher's exact test.

^c^ Compared by the post hoc Dunn tests (Bonferroni correction).

^d^ Compared by the pairwise comparisons with Bonferroni‐adjusted *p* values.

### 3.2 Validation of Propensity Score Weighting

The primary analysis comparing the aspirin‐continuation group (Group 1) and the control group (Group 3) utilized IPW to balance the significant baseline differences. The propensity score model demonstrated moderate discriminatory ability, with an area under the receiver operating characteristic curve (AUC) of 0.723 (95% confidence interval [CI], 0.670–0.776) (Figure S1). After applying IPW, a satisfactory balance across the baseline covariates was achieved. As detailed in Table 2, all SMDs were reduced to below the prespecified threshold of 0.1, except for “complex segmentectomy” (SMD = 0.131). The improved balance of the propensity score distributions after weighting is also visually represented in Figure S2.

**TABLE 2:** Covariate balance before and after the inverse probability of weighting.

Covariates	Standard mean difference	
	Unadjusted	IPW‐adjusted
Age		
≥ 80 years old	0.258	0.054
Sex		
Male	0.521	−0.054
BMI		
≥ 30	−0.067	−0.091
Smoking history		
Yes	0.705	−0.000
VC		
< 80% predicted	0.094	−0.015
FEV_1.0_/FVC		
< 70% predicted	0.189	0.032
Interstitial pneumonia		
Yes	0.378	0.003
Surgical approach		
VATS	0.215	−0.093
Operative procedure		
Lobectomy	0.168	0.026
Complex segmentectomy		
Yes	−0.015	0.131
Concomitant lung resection		
Yes	−0.151	−0.078
Resected side		
Right	−0.031	0.057
Resected lobe		
Upper	−0.006	0.059
Nodal dissection		
Hilar + mediastinal LNs	0.021	0.040
Whole pleural adhesion		
Yes	0.141	−0.010
Incomplete pulmonary fissure		
Yes	0.095	−0.035

Abbreviations: BMI, body mass index; FEV_1_, forced expiratory volume in 1 s; FVC, forced vital capacity; IPW, inverse probability weighting; LNs, lymph nodes; VATS, video‐assisted thoracoscopic surgery; VC, vital capacity.

### 3.3 Perioperative Outcomes (IPW‐Adjusted Analysis)

Unadjusted outcomes for all three groups are summarized in Table S2. Notably, the discontinuation group had a higher incidence of adverse events than the other groups, with a MACCE rate of 7.9% and a 90‐day mortality rate of 4.8%. In contrast, IPW‐adjusted comparisons of primary and secondary outcomes between the aspirin‐continuation group and the Control group are presented in Table 3. For the primary endpoints, no statistically significant difference was observed in the incidence of major bleeding (odds ratio [OR], 5.87; 95% CI, 0.80–42.91; *p* = 0.08) or MACCE (OR, 1.51; 95% CI, 0.35–6.45; *p* = 0.58). Regarding the secondary outcomes, the IPW‐adjusted analysis found no significant differences in intraoperative blood loss ≥ 100 mL, operative duration ≥ 300 min, conversion to open thoracotomy, or any complication graded ≥ II by the Clavien–Dindo classification.

**TABLE 3:** IPW‐adjusted comparison of primary and secondary outcomes between the aspirin‐continuation and control groups.

Perioperative outcomes	Unadjusted OR	95% CI	*p*	IPW‐adjusted OR	95% CI	*p*
Major bleeding	3.37	0.56–20.45	0.19	5.87	0.80–42.91	0.08
Reoperation for bleeding	2.51	0.22–27.92	0.46	1.70	0.15–19.24	0.67
RBC transfusion of ≥ 2 units within 48 h	2.51	0.22–27.92	0.46	6.69	0.59–75.66	0.13
Bleeding leading to death	1 (omitted)	—	—	1 (omitted)	—	—
MACCE	2.54	0.62–10.34	0.19	1.51	0.35–6.45	0.58
All‐cause mortality	1 (omitted)	—	—	1 (omitted)	—	—
Nonfatal myocardial infarction	1 (omitted)	—	—	1 (omitted)	—	—
Nonfatal ischemic stroke	5.07	0.70–36.42	0.11	3.28	0.44–24.69	0.25
Other surgical outcomes						
≥ 100 mL intraoperative blood loss	2.02	0.94–4.35	0.07	1.86	0.75–4.65	0.18
≥ 300 min of operative duration	1.60	0.97–2.65	0.07	1.19	0.65–2.18	0.58
Conversion to open thoracotomy for any reason	1.67	0.33–8.42	0.53	3.52	0.65–19.09	0.14
Reoperation due to any reason	0.35	0.05–2.68	0.31	0.26	0.03–2.06	0.20
Any complication grade ≥ II by the Clavien–Dindo classification	1.55	0.91–2.65	0.11	0.99	0.54–1.81	0.98
Atelectasis	0.99	0.11–8.62	1.00	0.49	0.06–4.24	0.51
Acute exacerbation of interstitial pneumonia	1 (omitted)	—	—	1 (omitted)	—	—
Acute heart failure	1 (omitted)	—	—	1 (omitted)	—	—
Atrial arrhythmia	1.34	0.44–4.14	0.61	1.01	0.31–3.28	0.99
Chylothorax	1.25	0.14–11.29	0.84	0.80	0.09–7.36	0.85
Pleural empyema	1.11	0.24–5.22	0.90	0.79	0.14–4.56	0.79
Pneumonia	2.54	0.62–10.34	0.19	1.88	0.44–8.01	0.39
Prolonged air leakage	0.44	0.10–1.91	0.27	0.25	0.05–1.19	0.08
Respiratory failure	5.02	0.31–81.00	0.26	3.77	0.23–61.29	0.35
Urinary tract infection	5.07	0.70–36.42	0.11	3.57	0.49–26.10	0.21
Wound infection	2.04	0.62–6.63	0.24	1.32	0.39–4.44	0.66

Abbreviations: CI, confidence interval; IPW, inverse probability weighting; MACCE, major adverse cardiac and cerebrovascular events; OR, odds ratio; RBC, red blood cell.

### 3.4 Predictors of Intraoperative Blood Loss

A multivariable linear regression analysis was performed to identify independent predictors of intraoperative blood loss among patients in the Aspirin‐continuation and Control groups (Table 4). In the IPW‐adjusted multivariable model, perioperative aspirin continuation was not identified as an independent predictor of increased intraoperative blood loss (*β* = 43.89; 95% CI, −31.09 to 118.87; *p* = 0.25). In contrast, the unadjusted model had suggested a significant association (*p* = 0.03).

**TABLE 4:** Linear regression analysis for predictors of intraoperative blood loss.

Variables	Unadjusted						IPW‐adjusted					
	Univariate			Multivariate			Univariate			Multivariate		
	*β* coefficient	95% CI	*p*	*β* coefficient	95% CI	*p*	*β* coefficient	95% CI	*p*	*β* coefficient	95% CI	*p*
Age												
≥ 80 years old	17.22	−0.15 to 35.60	0.05	3.43	−13.89 to 20.75	0.70	−8.76	−57.89 to 40.37	0.73			
Sex												
Male	8.49	−3.82 to 20.79	0.18	4.22	−11.22 to 19.66	0.59	−34.62	−119.86 to 50.62	0.43			
BMI												
≥ 30	31.33	−4.14 to 66.80	0.08	35.13	0.74–69.52	0.05	3.15	−61.33 to 67.62	0.92			
Smoking history												
Yes	10.77	−1.99 to 23.53	0.10	0.54	−15.63 to 16.71	0.95	−39.18	−145.48 to 67.11	0.47			
VC												
< 80% predicted	15.45	−13.52 to 44.14	0.30				0.53	−45.39 to 46.45	0.98			
FEV_1.0_/FVC												
< 70%	10.79	−1.51 to 23.09	0.09	5.63	−6.85 to 18.12	0.38	51.59	−33.57 to 136.76	0.24			
Interstitial pneumonia												
Yes	−3.11	−32.66 to 26.43	0.84				−22.73	−64.75 to 19.28	0.29			
Surgical approach												
VATS	8.66	−9.45 to 26.76	0.35				19.32	−33.05 to 71.69	0.47			
Operative procedure												
Lobectomy	24.21	10.50–37.93	0.00	12.50	−5.33 to 30.34	0.17	46.80	−3.45 to 97.04	0.07	34.87	−4.87 to 74.60	0.09
Complex segmentectomy												
Yes	−22.05	−40.64 to 3.46	0.02	−6.64	−29.78 to 16.50	0.57	−40.67	−84.68 to 3.34	0.07	−12.47	−36.09 to 11.15	0.30
Concomitant lung resection												
Yes	−13.54	−43.06 to 15.98	0.37				−35.75	−75.21 to 3.71	0.08	−40.53	−93.64 to 12.59	0.14
Resected side												
Right	9.48	−2.97 to 21.94	0.14	4.15	−8.31 to 16.61	0.51	−34.68	−132.19 to 62.83	0.49			
Resected lobe												
Upper	12.80	−0.03 to 25.63	0.05	10.50	−2.02 to 23.02	0.10	40.71	−14.80 to 96.22	0.15	40.45	−16.49 to 97.39	0.16
Nodal dissection												
Hilar + mediastinal LNs	4.05	−8.23 to 16.33	0.52				31.85	−33.36 to 97.06	0.34			
Whole pleural adhesion												
Yes	112.49	78.18–146.81	< 0.001	105.49	70.53–140.46	< 0.001	146.38	35.60–257.16	0.01	132.52	19.48–245.57	0.02
Incomplete pulmonary fissure												
Yes	5.41	−8.33 to 19.14	0.44				−56.20	−187.95 to 75.56	0.40			
Perioperative aspirin continuation												
Yes	23.49	7.29–39.70	0.01	18.10	1.97–34.24	0.03	43.89	−31.09 to 118.87	0.25			

Abbreviations: BMI, body mass index; CI, confidence interval; FEV_1_, forced expiratory volume in 1 s; FVC, forced vital capacity; IPW, inverse probability weighting; LNs, lymph nodes; VATS, video‐assisted thoracoscopic surgery; VC, vital capacity.

### 3.5 Subgroup Analysis Within the Aspirin‐Continuation Group

A subgroup analysis was performed within the aspirin‐continuation group (Group 1) to compare outcomes between patients who continued chronic aspirin monotherapy (*n* = 70) and those who were switched from clopidogrel to aspirin perioperatively (*n* = 24). The results are presented in Table S3. There were no statistically significant differences between these two subgroups in the incidence of major bleeding, MACCE, intraoperative blood loss, or any other primary or secondary endpoint measured.

## 4 Discussion

This single‐center, retrospective study quantified the risk of a perioperative aspirin‐continuation strategy in high‐risk, secondary‐prevention patients undergoing anatomical lung resection. We used IPW to compare 94 patients in the Aspirin‐continuation group with 468 in the Control group. The primary IPW‐adjusted analysis revealed no significant increase in major bleeding or MACCE. The adjusted secondary multivariable analysis also did not identify aspirin as an independent predictor of increased intraoperative blood loss.

This study's novelty is its robust IPW adjustment for both primary endpoints and the multivariable blood loss analysis. Our IPW‐adjusted analysis (unlike the unadjusted) found no aspirin‐related increase in intraoperative blood loss. This finding strengthens our primary result, indicating that aspirin did not increase major bleeding or overall blood loss when balanced. This result aligns with previous studies that also reported no increase in blood loss, though many lacked propensity score adjustment for this outcome [6, 7, 8]. While Sakai et al. used PSM in VATS patients [9], our study used IPW on the entire cohort, compared against a no‐therapy control, and included converted thoracotomy cases, enhancing generalizability.

The consistent finding across thoracic surgery studies, including ours, that aspirin does not significantly increase catastrophic bleeding contrasts with the POISE‐2 trial, which advised against it due to major bleeding [3]. This divergence is likely attributable to different study populations: POISE‐2 was dominated by primary‐prevention patients, whereas our cohort was entirely high‐risk secondary prevention. This distinction is critical, as a POISE‐2 substudy showed aspirin reduced ischemic events specifically among high‐risk patients with prior percutaneous coronary intervention [10].

This consistency suggests that, in the modern context of minimally invasive procedures, the risk of aspirin‐related bleeding may be clinically manageable. This finding may be partly explained by modern thoracic surgery; our cohort consisted primarily of minimally invasive procedures or conversions (excluding planned open thoracotomy), in which advances in energy devices enable meticulous hemostasis [11]. Furthermore, unlike orthopedic or hepatic surgery with bone bleeding or large raw surfaces [12], critical hemorrhage in anatomical lung resection originates from major vessel stumps. These sites are definitely secured with staples or ligation, making them less susceptible to the diffuse microvascular oozing that aspirin provokes. While this may explain the lack of increased bleeding, this interpretation requires caution. The nonsignificant result for major bleeding (OR, 5.87; 95% CI, 0.80–42.91) is not definitive proof of safety. The extensive CI indicates the analysis was statistically underpowered, leaving the actual effect highly uncertain. The nonsignificant MACCE finding was similarly limited.

A key feature is our definition of the intervention group. The ‘Aspirin‐continuation group’ included stable chronic aspirin users and, critically, high‐risk patients (25.5%) switched from clopidogrel perioperatively, reflecting a common, high‐stakes scenario. Our subgroup analysis revealed no significant differences in outcomes between these chronic users and the clopidogrel‐switch subgroup. While small sample sizes limited this analysis, the lack of a detectable difference suggests the “aspirin‐bridging” strategy did not confer a substantially increased risk compared to stable aspirin monotherapy.

Furthermore, although the Antiplatelet‐ and anticoagulant‐discontinuation group (Group 2) was excluded from the IPW analysis due to significant heterogeneity, its descriptive data provide critical clinical insights. Although the analysis for Group 2 was purely descriptive, the markedly high rates of MACCE (7.9%) and 90‐day mortality (4.8%) observed in this group provide critical clinical insights. While these findings may be influenced by the patients' inherent high‐risk baseline characteristics, they align with existing evidence suggesting that discontinuing secondary prevention carries a substantial thrombotic risk [13, 14]. This observation underscores the clinical rationale for our ‘aspirin‐continuation’ strategy; the potential for a nonsignificant trend toward increased bleeding may be a necessary trade‐off to avoid the catastrophic thromboembolic consequences of therapy interruption.

This study has several crucial limitations. First, its retrospective, single‐center design is susceptible to selection bias. Although IPW was used to mitigate this by balancing measured covariates, it cannot account for unmeasured confounders, such as surgeon preference or patient frailty. Second, despite IPW adjustment, a minor residual imbalance remained in the “complex segmentectomy” variable (SMD = 0.131), exceeding our prespecified threshold. We retained this model because it provided the best overall balance for critical clinical confounders. Third, and most critically, the study was statistically underpowered to detect differences in the rare primary endpoints of major bleeding and MACCE, given the wide CIs. Fourth, the Aspirin‐continuation group was heterogeneous, including both chronic aspirin users and patients switched from clopidogrel. Although our subgroup analysis did not detect differences in outcomes, it was itself significantly underpowered (*n* = 24 in the switch subgroup), precluding definitive conclusions regarding the safety of this “aspirin‐bridging” strategy. Finally, our exclusion of planned open thoracotomy limits the generalizability of these findings, as they primarily apply to minimally invasive procedures.

## 5 Conclusions

Although perioperative aspirin continuation did not reach statistical significance for increased major bleeding or MACCE, a trend toward increased bleeding risk was observed. Given limited statistical power and wide CIs, the safety of this strategy remains unproven. However, our descriptive data on therapy discontinuation underscore the substantial risk of thromboembolic events. Therefore, the decision to continue aspirin must be a carefully weighed clinical judgment, balancing individual thrombotic benefits against the potential for increased surgical bleeding.

## Author Contributions

**Toshiki Tanaka:** supervision, data curation, writing – review and editing. **Junichi Murakami:** conceptualization, methodology, validation, formal analysis, project administration, writing – original draft, visualization, investigation. **Sota Yoshimine:** conceptualization, data curation, writing – review and editing, validation, investigation. **Mototsugu Shimokawa:** validation, formal analysis, writing – review and editing.

## Funding

The authors have nothing to report.

## Conflicts of Interest

The authors declare no conflicts of interest.

## Supporting information


**Figure S1:** Receiver operating characteristic (ROC) curve for the propensity score model. The propensity score model for distinguishing between the aspirin‐continuation group and the control group yielded an area under the curve (AUC) of 0.723 (95% CI, 0.670–0.776), indicating moderate discrimination.
**Figure S2:** Distribution of propensity scores before and after the inverse probability weighting. The density plots show the distributions of propensity scores for the aspirin‐continuation group (Group 1) and the control group (Group 3) before (raw) and after (weighted) inverse probability weighting (IPW).
**Table S1:** Details of preoperative antithrombotic agents by group.
**Table S2:** Unadjusted perioperative outcomes among the three groups.
**Table S3:** Subgroup analysis of outcomes within the aspirin‐continuation group (Group 1).

## Data Availability

The data that support the findings of this study are available from the corresponding author upon reasonable request.
