# Supplementary material for: Perioperative Aspirin Continuation in Patients Requiring Secondary Prevention Undergoing Anatomical Lung Resection: A Propensity Score‐Weighted Analysis
Source: Thorac Cancer. 2026 Jul 21;17(14):e70364. doi: 10.1111/1759-7714.70364 (PMC13389234; doi:10.1111/1759-7714.70364)
Supplement: Supplementary file 1 — Figure S1: Receiver operating characteristic (ROC) curve for the propensity score model. The propensity score model for distinguishing between the aspirin‐continuation group and the control group yielded an area under the curve (AUC) of 0.723 (95% CI, 0.670–0.776), indicating moderate discrimination. Figure S2: Distribution of propensity scores before and after the inverse probability weighting. The density plots show the distributions of propensity scores for the aspirin‐continuation group (Group 1) and the control group (Group 3) before (raw) and after (weighted) inverse probability weighting (IPW). Table S1: Details of preoperative antithrombotic agents by group. Table S2: Unadjusted perioperative outcomes among the three groups. Table S3: Subgroup analysis of outcomes within the aspirin‐continuation group (Group 1). [file TCA-17-e70364-s001.docx]

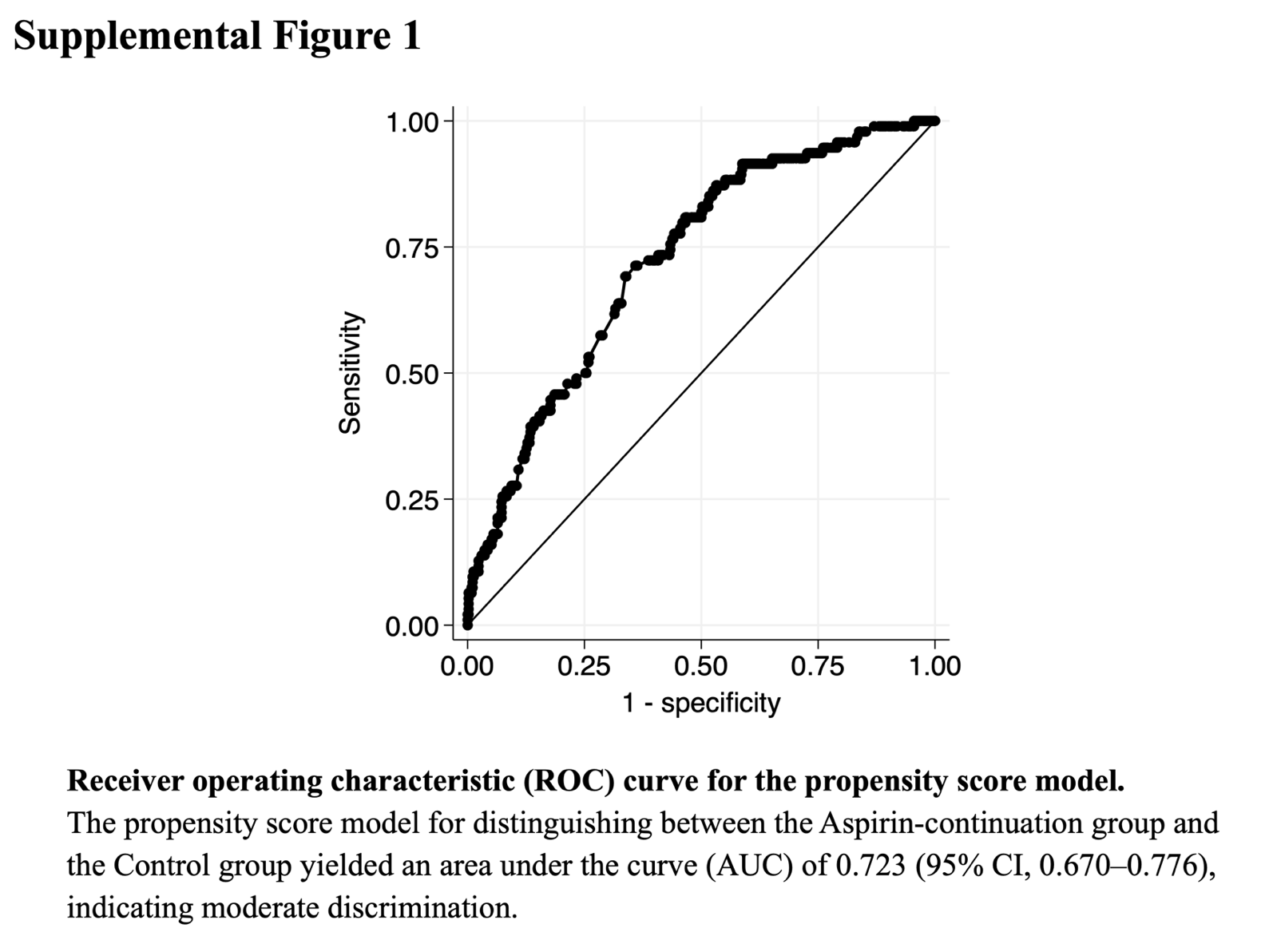


**Receiver operating characteristic (ROC) curve for the propensity score model.**

The propensity score model for distinguishing between the Aspirin-continuation group and the Control group yielded an area under the curve (AUC) of 0.723 (95% CI, 0.670-0.776), indicating moderate discrimination.


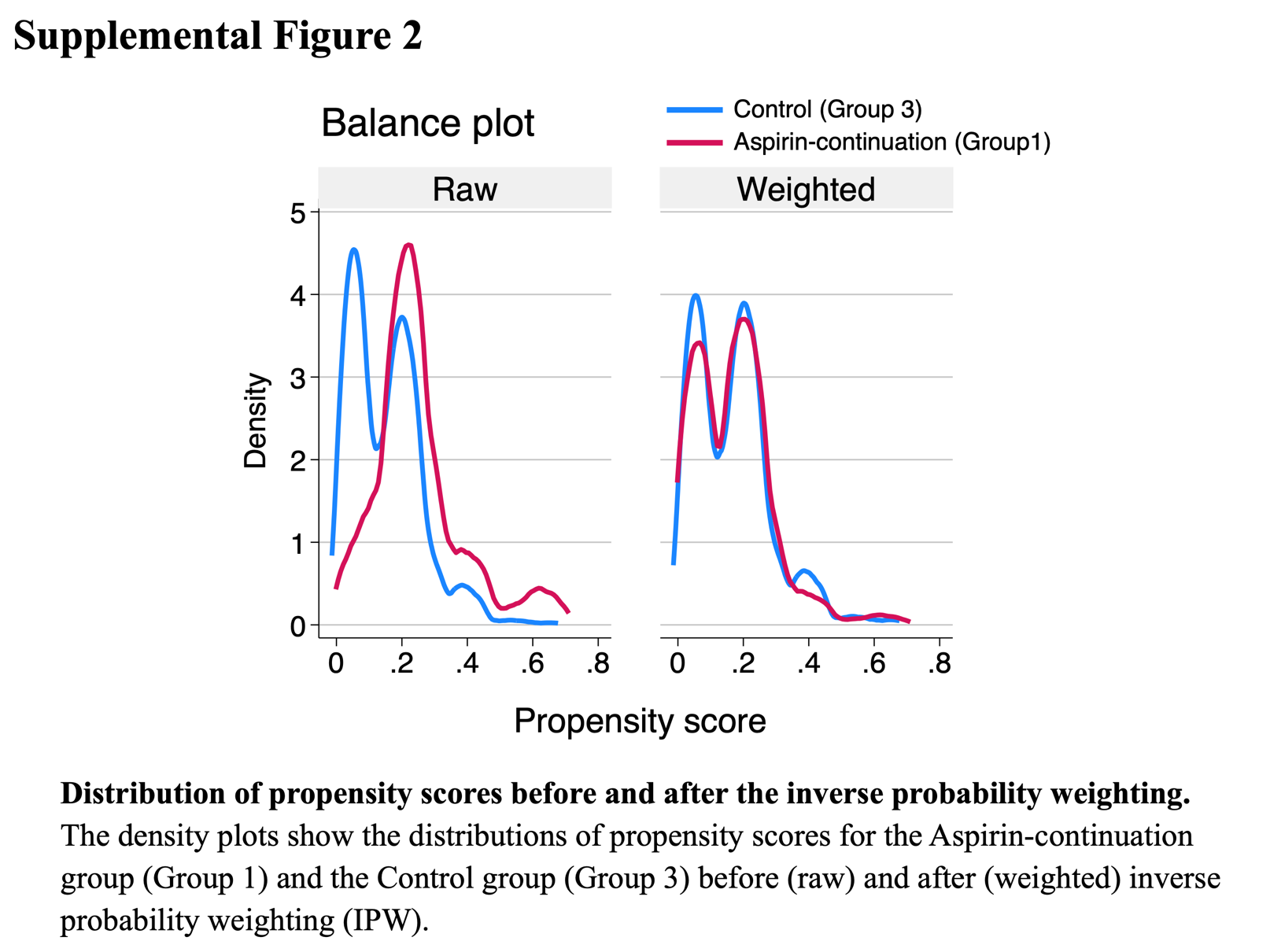


**Distribution of propensity scores before and after the inverse probability weighting.**

The density plots show the distributions of propensity scores for the Aspirin-continuation group (Group 1) and the Control group (Group 3) before (raw) and after (weighted) inverse probability weighting (IPW).

| **Supplemental Table 1.** **Details of preoperative antithrombotic agents by group.** | | | | |  |  |
| --- | --- | --- | --- | --- | --- | --- |
|  | **Group 1** |  | **Group 2** |  | **Group 3** |  |
| **Variables** | **Aspirin-continuation** | | **Antiplatelet and Anticoagulant Discontinuation** | | **Control** |  |
|  | n = 94 (%) |  | n = 63 (%) |  | n = 468 (%) |  |
| **Antipeletet** | 94 (100.0) | (100.0) | 26 (41.3) | 41.3 | 0 (0.0) | (0.0) |
| Aspirin | 70 (74.5) | 74.5 | 0 (0.0) | (0.0) | 0 (0.0) | (0.0) |
| Cilostazol | 4 (4.3) | 4.3 | 15 (23.8) | 23.8 | 0 (0.0) | (0.0) |
| Clopidogrel | 24 (25.5) | 25.5 | 9 (14.3) | 14.3 | 0 (0.0) | (0.0) |
| Prasugrel | 6 (6.4) | 6.4 | 1 (1.6) | 1.6 | 0 (0.0) | (0.0) |
| Sarpogrelate | 0 (0.0) | (0.0) | 2 (3.2) | 3.2 | 0 (0.0) | (0.0) |
| **Anticoaglant** | 7 (7.5) | 7.5 | 38 (60.3) | 60.3 | 0 (0.0) | (0.0) |
| Apixaban | 1 (1.1) | 1.1 | 11 (17.5) | 17.5 | 0 (0.0) | (0.0) |
| Dabigatran | 0 (0.0) | 0 | 3 (4.8) | 4.8 | 0 (0.0) | (0.0) |
| Edoxaban | 2 (2.1) | 2.1 | 15 (23.8) | 23.8 | 0 (0.0) | (0.0) |
| Rivaroxaban | 2 (2.1) | 2.1 | 1 (1.6) | 1.6 | 0 (0.0) | (0.0) |
| Warfarin | 2 (2.1) | 2.1 | 8 (12.7) | 12.7 | 0 (0.0) | (0.0) |

Preoperative antithrombotic agents in the Aspirin-continuation group (Group 1) and the Antiplatelet- and anticoagulant-discontinuation group (Group 2).

| **Supplemental Table 2.** **Unadjusted perioperative outcomes among the three groups.** | | | | | |
| --- | --- | --- | --- | --- | --- |
| Variables | **All patients** | **Group 1** | **Group 2** | **Group 3** | ***P*-value** |
|  |  | **Aspirin-continuation** | **Antiplatelet and Anticoagulant Discontinuation** | **Control** |  |
|  | n = 625, (%), [95% CI] | n = 94, (%),  [95% CI] | n = 63, (%),  [95% CI] | n = 468, (%),  [95% CI] |  |
| **Major bleeding** | 6 (1.0) | 2 (2.1) | 1 (1.6) | 3 (0.6) | 0.22^b^ |
| Reoperation for bleeding | 3 (0.5) | 1 (1.1) | 0 (0.0) | 2 (0.4) | 0.58^b^ |
| RBC transfusion of ≥4 units within 48 hours | 4 (0.6) | 1 (1.1) | 1 (1.6) | 2 (0.4) | 0.26^b^ |
| Bleeding leading to death | 0 (0.0) | 0 (0.0) | 0 (0.0) | 0 (0.0) | - |
| **MACCE** | 14 (2.2) | 3 (3.2) | 5 (7.9) | 6 (1.3) | 0.006^b^ |
| All-cause mortality | 7 (1.1) | 0 (0.0) | 3 (4.8) | 4 (0.9) | 0.04^b^ |
| Non-fatal myocardial infarction | 2 (0.3) | 1 (1.1) | 1 (1.6) | 0 (0.0) | 0.06^b^ |
| Non-fatal ischemic stroke | 6 (1.0) | 2 (2.1) | 2 (3.2) | 2 (0.4) | 0.04^b^ |
| **Intraoperative blood loss (mL)** | 20 [10−30] | 20 [15−50] | 20 [15−45] | 15 [10−30] | <0.001^a^ |
| ≥100 mL blood loss | 41 (6.6) | 10 (10.6) | 5 (7.9) | 26 (5.9) | 0.16^b^ |
| ≥10% blood volume loss | 3 (0.5) | 3 (3.2) | 0 (0.0) | 2 (0.4) | 0.04^b^ |
| **Operative duration (minutes)** | 247 [206−294] | 251 [218−306] | 259 [212−308] | 244 [203−244] | 0.04^a^ |
| ≥300 minutes of operative duration | 138 (22.1) | 27 (28.7) | 17 (27.0) | 94 (20.1) | 0.11^b^ |
| **Conversion to open thoracotomy due to any reasons** | 10 (1.6) | 2 (2.1) | 2 (3.2) | 6 (1.3) | 0.29^b^ |
| **Reoperation due to any reasons** | 18 (2.9) | 1 (1.1) | 3 (4.8) | 14 (3.0) | 0.39^b^ |
| **Any complication graded ≥II**  **by the Clavien-Dindo classification** | 119 (19.0) | 22 (23.4) | 20 (31.8) | 77 (16.5) | 0.009^b^ |
| Atelectasis | 6 (1.0) | 1 (1.1) | 0 (0.0) | 5 (1.1) | 1.00^b^ |
| Acute exacerbation of interstitial pneumonia | 4 (0.6) | 0 (0.0) | 2 (3.2) | 2 (0.4) | 0.09^b^ |
| Acute heart failure | 2 (0.3) | 1 (1.1) | 1 (1.6) | 0 (0.0) | 0.06^b^ |
| Atrial arrhythmia | 22 (3.5) | 4 (4.3) | 3 (4.8) | 15 (3.5) | 0.57^b^ |
| Chylothorax | 5 (0.8) | 1 (1.1) | 0 (0.0) | 4 (0.9) | 1.00^b^ |
| Pleural empyema | 14 (2.2) | 2 (2.1) | 3 (4.8) | 9 (1.9) | 0.33^b^ |
| Pneumonia | 12 (1.9) | 3 (3.2) | 3 (4.8) | 6 (1.3) | 0.06^b^ |
| Prolonged air leakage | 30 (4.8) | 2 (2.1) | 6 (9.5) | 22 (4.7) | 0.11^b^ |
| Respiratory failure | 3 (0.5) | 1 (1.1) | 1 (1.6) | 1 (0.2) | 0.16^b^ |
| Wound infection | 14 (2.2) | 4 (4.3) | 0 (0.0) | 10 (2.1) | 0.20^b^ |
| Urinary tract infection | 4 (0.6) | 2 (2.1) | 0 (0.0) | 2 (0.4) | 0.16^b^ |
| Other complications | 29 (2.6) | 29 (2.6) | 13 (0.0) | 17 (2.1) | 0.02^b^ |
| **Duration of chest tube drainage** | 1 [1−1] | 1 [1−1] | 1 [1−1] | 1 [1−1] | 0.41^a^ |
| **Length of hospital stay** | 5 [4−6] | 5 [5−8] | 5 [5−9] | 4 [5−6] | <0.001^a^ |
| **30 days mortality** | 2 (0.3) | 0 (0.0) | 1 (1.6) | 1 (0.2) | 0.21^b^ |
| **90 days mortality** | 7 (1.1) | 0 (0.0) | 3 (4.8) | 4 (4.8) | 0.04^b^ |
| Values are expressed as number (%) or median (interquartile range [IQR]). a. compared by the Kruskal-Wallis test, b. compared using the chi-square test or Fisher's exact test. CI; confidence Interval, RBC; red blood cell, MACCE; major adverse cardiac and cerebrovascular events. | | | | | |

Unadjusted perioperative outcomes across the three study groups (Group 1, Aspirin-continuation; Group 2, Discontinuation; Group 3, No-therapy controls).

| **Supplemental Table 3.** **Subgroup analysis of outcomes within the Aspirin-continuation group (Group 1).** | | | | |
| --- | --- | --- | --- | --- |
| Variables | **Group 1** | | | ***P*-value** |
|  | **Total** | **Aspirin Monotherapy Continuation subgroup** | **Clopidogrel Switch subgroup** |  |
|  | n = 94, (%), [95% CI] | n = 70, (%), [95% CI] | n = 24, (%), [95% CI] |  |
| **Major bleeding** | 2 (2.1) | 2 (2.9) | 0 (0.0) | 1.00^b^ |
| Reoperation for bleeding | 1 (1.1) | 1 (1.4) | 0 (0.0) | 1.00^b^ |
| RBC transfusion of ≥4 units within 48 hours | 1 (1.1) | 1 (1.4) | 0 (0.0) | 1.00^b^ |
| Bleeding leading to death | 0 (0.0) | 0 (0.0) | 0 (0.0) | - |
| **MACCE** | 3 (3.2) | 2 (2.9) | 1 (4.2) | 1.00^b^ |
| All-cause mortality | 0 (0.0) | 0 (0.0) | 0 (0.0) | - |
| Non-fatal myocardial infarction | 1 (1.1) | 1 (1.5) | 0 (0.0) | 1.00^b^ |
| Non-fatal ischemic stroke | 2 (2.1) | 1 (1.5) | 1 (4.2) | 0.45^b^ |
| **Intraoperative blood loss (mL)** | 20 [15−50] | 20 [15−45] | 20 [10−62] | 0.53^a^ |
| ≥100 mL blood loss | 10 (10.6) | 8 (11.4) | 2 (8.3) | 1.00^b^ |
| ≥10% blood volume loss | 3 (3.2) | 3 (4.3) | 0 (0.0) | 0.57^b^ |
| **Operative duration (minutes)** | 251 [218−306] | 251 [212−314] | 259 [223−300] | 0.88^a^ |
| ≥300 minutes of operative duration | 27 (28.7) | 21 (30.0) | 6 (25.0) | 0.80^b^ |
| **Conversion to open thoracotomy due to any reasons** | 2 (2.1) | 2 (2.9) | 0 (0.0) | 1.00^b^ |
| **Reoperation due to any reasons** | 1 (1.1) | 1 (1.4) | 0 (0.0) | 1.00^b^ |
| **Any complication graded ≥II**  **by the Clavien-Dindo classification** | 22 (23.4) | 16 (22.9) | 6 (25.0) | 0.79^b^ |
| **Duration of chest tube drainage** | 1 [1−1] | 1 [1−1] | 1 [1−1] | 0.81^a^ |
| **Length of hospital stay** | 5 [5−8] | 6 [5−8] | 5 [5−7] | 0.27^a^ |
| **30 days mortality** | 0 (0.0) | 0 (0.0) | 0 (0.0) | - |
| **90 days mortality** | 0 (0.0) | 0 (0.0) | 0 (0.0) | - |
| Aspirin Monotherapy Continuation subgroup: Patients who continued chronic aspirin alone, or were on aspirin plus another antiplatelet agent(s) preoperatively and continued only aspirin perioperatively.Clopidogrel Switch subgroup: Patients who were switched from chronic clopidogrel to aspirin specifically for the perioperative period. Values are expressed as number (%) or median (interquartile range [IQR]). a; compared by the Wilcoxon rank sum test, b; compared using the chi-square test or Fisher's exact test. CI; confidence Interval, RBC; red blood cell, MACCE; major adverse cardiac and cerebrovascular events. | | | | |

Subgroup analysis within the Aspirin-continuation group (Group 1) comparing patients who continued chronic aspirin monotherapy (n = 70) with those switched from clopidogrel to aspirin perioperatively (n = 24).
